# Anti Transglutaminase Antibodies Cause Ataxia in Mice

**DOI:** 10.1371/journal.pone.0009698

**Published:** 2010-03-15

**Authors:** Sabrina Boscolo, Andrea Lorenzon, Daniele Sblattero, Fiorella Florian, Marco Stebel, Roberto Marzari, Tarcisio Not, Daniel Aeschlimann, Alessandro Ventura, Marios Hadjivassiliou, Enrico Tongiorgi

**Affiliations:** 1 Department of Life Sciences, University of Trieste, Trieste, Italy; 2 CSPA, University of Trieste, Trieste, Italy; 3 Department of Medical Science and IRCAD, University of Eastern Piedmont, Novara, Italy; 4 Institute for Childhood Disease, IRCCS Burlo Garofolo, Department of Reproductive and Development Science, University of Trieste, Trieste, Italy; 5 Matrix Biology and Tissue Repair Research Unit, School of Dentistry, Cardiff University, Cardiff, United Kingdom; 6 Department of Clinical Neurology, Royal Hallamshire Hospital, Sheffield, United Kingdom; 7 BRAIN Centre for Neuroscience, Department of Life Sciences, University of Trieste, Trieste, Italy; Julius-Maximilians-Universität Würzburg, Germany

## Abstract

**Background:**

Celiac disease (CD) is an autoimmune gastrointestinal disorder characterized by the presence of anti-transglutaminase 2 (TG2) and anti-gliadin antibodies. Amongst the neurological dysfunctions associated with CD, ataxia represents the most common one.

**Methods:**

We analyzed by immunohistochemistry, the anti-neural reactivity of the serum from 20 CD patients. To determine the role of anti-TG2 antibodies in ataxia, two anti-TG2 single chain variable fragments (scFv), isolated from a phage-display IgA antibody library, were characterized by immunohistochemistry and ELISA, and injected in mice to study their effects on motor coordination. We found that 75% of the CD patient population without evidence of neurological involvement, has circulating anti-neural IgA and/or IgG antibodies. Two anti-TG2 scFvs, cloned from one CD patient, stained blood vessels but only one reacted with neurons. This anti-TG2 antibody showed cross reactivity with the transglutaminase isozymes TG3 and TG6. Intraventricular injection of the anti-TG2 or the anti-TG2/3/6 cross-reactive scFv provoked transient, equally intensive ataxia in mice.

**Conclusion:**

The serum from CD patients contains anti-TG2, TG3 and TG6 antibodies that may potentially cause ataxia.

## Introduction

Gluten sensitive enteropathy or celiac disease (CD) is a common (1 in 90) state of heightened immunological responsiveness to the ingestion of gluten in genetically predisposed individuals having the HLA class II haplotype DQ2 or DQ8 [1]. The clinical spectrum of CD ranges from asymptomatic to the classic malabsorption syndrome [2]. In addition, a variety of neurological manifestations have been identified, including ataxia [3], epilepsy [4], brain atrophy, headache with white matter lesions and polyneuropathy [5].

The serum from patients with CD contains characteristic antibodies against the self antigen, tissue transglutaminase (TG2) [6] as well as against gliadin, the most abundant gluten protein. In addition, in the effector phase of the disease the epithelium of the small intestine contains infiltrates of TCRαβ+ CD8+ T cells proportional to the tissue damage [7], [8], [9]. Furthermore, other autoimmune diseases are often present in patients with CD or their first-degree relatives sharing the same HLA haplotype [10].

It has been hypothesized that ataxia seen in the context of gluten sensitivity, also called gluten ataxia, might be caused by an autoimmune mechanism. This hypothesis is supported by the finding that in some patients IVIg therapy succeeded [11], [12], [13] and that serum from patients with gluten ataxia reacts with Purkinje cells. This reactivity has been attributed in part to circulating anti-gliadin antibodies and in part to other, yet unidentified, antibodies [14]. A pathogenic role of these antibodies is suggested by the fact that intraventricular injections of serum from patients with gluten ataxia cause a transient ataxia in mice. Injection of sera from patients with CD without neurological complications however, has similar effects [15]. These data suggest that serum antibodies from CD patients might have an intrinsic potential to induce ataxia, and possibly neurodegeneration, when they enter the CNS.

Anti-TG2 antibodies, the specific marker of CD, are primarily deposited locally in the intestine but they have also been found in liver, muscle and lymph nodes [16]. Recent studies also demonstrated intrathecal production of anti-TG2 antibodies in patients with neurological disease [17] and the presence of perivascular IgA deposits in the cerebellum of patients with gluten ataxia [18]. In the present study we investigated whether differential reaction of patient-derived anti-TG2 antibodies [8] with cerebellar neurons could explain the development of ataxia.

## Materials and Methods

### Sera

Sera were collected in the Transfusional centre of the IRCCS Burlo Garofolo Hospital of Trieste following written informed consent and approval by the Scientific Directorate. The investigation was approved by the Local Ethical Committee. Sera from twenty CD patients with no known neurological complications were collected at diagnosis of CD (mean age 35.4 yr; range 20–61 yr) and 20 donor sera were used as healthy controls (mean age 35.4 yr; range 23–60 yr). All CD patients sera were positive for anti-TG2, anti-gliadin and anti-EMA antibodies.

Anti-TG2 and anti-gliadin antibodies were measured with Eu-tTG or Alpha-gliatest kits (Eurospital, Trieste, Italy) respectively. Anti-endomysium antibodies (EMA) and islet cell autoantibodies (ICA) were detected by immunohistochemistry. Antibodies to mitochondrial antigens (AMA), to smooth muscle antigens (SMA) and to nuclear antigens (ANA) were detected with the Alphadia autoscreen II test system (Axis-Shield, Cambridgeshire, UK).

### Animals

Animals were housed at the University animal house according to institutional guidelines, and all animal work was approved by the Institutional Review Committee for Animal Studies of the University of Trieste in accordance with national (DL N116, GU, suppl 40, 18-12-1992) and international laws and policies (European Community Council Directive 86/609, December 12, 1987). Animals were purchased from Harlan Italy (San Pietro al Natisone, Italy), kept in individual cages in the Animal Facility of the University of Trieste in a controlled environment (T = 22°C, 12-/12-hour light/dark cycle), and fed a standard commercial chow diet (Harlan 2018, 3.4 kcal/g; Harlan). At the end of experiments animals were CO_2_ sacrificed, brains were immediately frozen in isopenthane/dry ice and stored at −80°C.

### Selection and purification of scFvs

Single chain (scFv) antibodies to TG2 were selected from a phage display antibody library on recombinant human TG2 (h-TG2) and based on analysis of reactivity with different TG2 fragments assigned to one of two groups named class1 and class2 [8]. cDNA amplification in library construction employed primers specific for c_H_1 heavy chain and the resulting library is consequently representative of a specific Ig class [19], IgA in this case. The control scFv named bac1 was selected against an unrelated E. coli protein from a large naïve scFvs library [20]. The scFvs are made up of linked v_L_ and v_H_ and contain both an His_6_ and a SV5 [21] tags. ScFv antibodies were purified by Ni-NTA chromatography (Qiagen, Milano, Italy), dialyzed against PBS and diluted to a final concentration of 0.1 mg/ml.

### Immunohistochemistry

Immunohistochemistry and densitometry on rat brain sections were carried out as previously described [22]. Immunohistochemistry was also performed on wild type and TG2^−/−^ C57Bl/6 male mice brains with both sera and monoclonal anti TG2 antibodies. Purified scFv antibodies (1∶1) were revealed with a secondary mouse anti-SV5 tag (1∶1) [21] and a tertiary HRP-conjugated anti-mouse IgG (1∶200, Jackson Immunoresearch labs, Suffolk, UK). TG2 was also detected with monoclonal antibody CUB7402 (LabVision, Cheshire, UK), (1∶1000). For preadsorption experiments, sera (1∶100 for IgA; 1∶600 for IgG) were incubated with 10 µg/ml of recombinant h-TG2, or 50 µg/ml of BSA as negative control, for 30 min at room temperature before use for immunohistochemistry.

### ELISA

Brain homogenates were prepared from C57Bl/6 wt, TG2^−/−^ mice, Sprague-Dawley rats, and an adult human Caucasian male in a tissue lysis buffer (25 mM TrisHCl pH 7.5, 1 mM EDTA, 1 mM spermidine, 1 mM phenylmethylsulfonylfluoride, 1 mM iodoacetamide, 1 mM soy bean trypsin inhibitor, 10 µg/ml turkey egg white inhibitor and 0,1%Triton X100). Antigens (brain homogenates or recombinant human TG2 [23], TG3 or TG6 [24] were diluted in PBS to 10 µg/ml immediately before use. Multi well strips (Costar, Corning Incorporated, NY, USA) were coated with 100 µl/well of the antigen solution (16 h, 4°C), washed with PBS containing 0.1%Tween20 and blocked with 200 µl/well of 2% skimmed milk in PBS for 1 h at room temperature. Strips were incubated for 1 h with scFvs (60 µg/ml), then with mouse anti-SV5 tag antibody [21] (1∶200) and finally with HRP-conjugated anti-mouse IgG (Jackson Immunoresearch labs, Suffolk, UK) (1∶1500). All antibodies were diluted in blocking solution (100 µl/well). Development was carried out with 1-step turbo TMB (Pierce, Rockford, Illinois, USA) and absorbance read at 450 nm.

### Motor coordination tests

Adult C57Bl/6 male mice were trained as described in supplemental material (Text S1) for motor coordination test on rotarod and their performance was subsequently evaluated following injection with scFvs.

Footprint analysis was performed on mice 5 h after injection with control or anti TG2 scFv antibodies class1 or class2. Atoxic and aqueous colors have been used (blood red and ultramarine blue, Citadel Paint)to paint paws. Mice instinctively run towards a closed box situated at the end of a corridor (50 cm length × 10 cm width) where a white paper is placed. Three test for each mouse were carried out and four different parameter considered: R-L forelimb stride and R-L hindlimb stride, frontbase, hindbase and finally R-L overlap. Four (class1 and bac1) or six (class2) mice for each injected antibody were tested and data considered only after detection of correct incannulation position with haematoxylin-eosin staining.

### Statistics

For comparison of reactivity of class1 or class2 scFvs on TG3 or TG6 in ELISAs, and for footprint analysis, one way ANOVA was used. For comparison between class1 or class2 scFv effect on mice balance for motor coordination test the Wilcoxon test was used.

### Localization of injected scFv

The purified scFvs class1, class2 or bac1 were biotinylated following the manufacturer's instructions (Pierce, Rockford, Illinois, USA), and 3 µl (100 ng/µl in PBS) were injected intraventricularly in mice. Three hours after injection mice were sacrificed. Cryosections of brains were fixed and endogenous peroxidase blocked with 0.3% H_2_O_2_ in methanol for 7 min, 0.3% H_2_O_2_ in 96% ethanol for 3 min, rehydrated in 0.3% H_2_O_2_ in 70% ethanol for 2 min, and rinsed with H_2_O and PBS. Biotinylated scFv was detected with HRP-conjugated streptavidin (1∶60,000 in PBS) (Pierce, Rockford, Illinois, USA) and revealed with the chemiluminescent detection system (ECL GE Healthcare, Milan, Italy). After injection, biotinilated scFvs were detected with FITC-conjugated streptavidin (1∶100 in PBS containing 0.05%Tween20) (Dako, Milano, Italy). Blood vessels were stained with anti-Von Willebrand factor (vWF) (1∶500 in PBS containing 0.05%Tween20) (Dako, Milano, Italy) followed by anti-rabbit conjugated with TRITC (1∶30 in PBS containing 0.05%Tween20) (Dako, Milano, Italy). Data were collected from 24 animals (8 per group).

## Results

### Clinical data

All 20 CD patients were positive for anti-TG2, EMA and AGA antibodies, were HLA-DQ2 or DQ8 positive and the intestinal biopsy showed villous atrophy, crypt hyperplasia and increased intraepithelial lymphocytes, in keeping with CD. Serological analysis for other autoimmunity markers (ICA, AMA, SMA, ANA) were negative. Healthy donors were negative for all autoantibodies tests and had no relatives presenting with autoimmune disorders.

### Anti-neural autoantibodies in patients with CD

**Figure 1:**
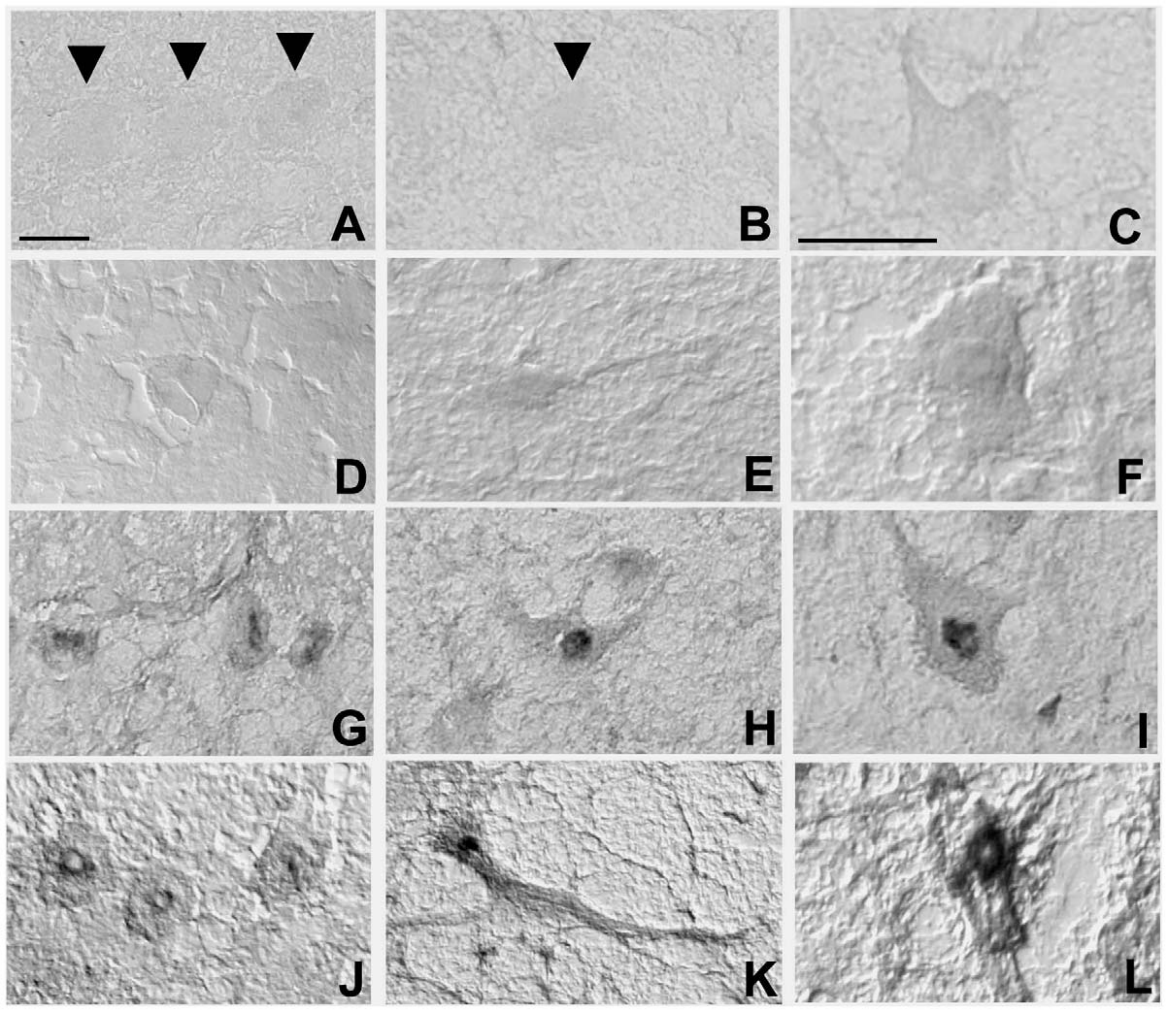
Reactivity of rat brain sections with serum IgAs and IgGs of CD patients. Immunohistochemical staining on rat Purkinje cells (left panels, arrow heads), deep cerebellar nuclei neurons (middle panels, arrow head) and brainstem reticular neurons (right panels) using either serum from a healthy donor (A–F) or from an untreated CD patient (G–L). No binding of serum IgAs (A–C) and IgGs (D–F) from healthy donors could be detected. In contrast, antibodies in sera of CD patient (positive for anti-TG2 IgA/IgG antibodies) stained the cytoplasm and the perinuclear zone of neurons from the three brain areas, both when detecting IgAs (G–I) or IgGs (J–L). Calibration bars = 20 µm.

Sera were tested for anti-neural immunoreactivity on rat brain sections by immunohistochemistry. Only three out of 20 healthy blood donor sera were positive (1 for both IgA and IgG, 2 for IgG only). Figure 1 shows a typical result for detection of IgA (Figure 1A, B, C) and IgG (Figure 1D, E, F), from a healthy subject on rat Purkinje cells, deep cerebellar nuclei and brainstem neurons. In contrast, 4 out of 20 patients with CD showed strong anti-neural IgA reactivity (Figure 1G, H, I) and 12 out of 20 had abundant immunoreactive IgG (Figure 1J, K, L). The most frequent staining pattern of sera from CD patients was characterized by a weak cytoplasmic and a strong perinuclear labeling (Figure 1G-L; Figure S1, CD1 and CD2). Sera from two patients stained only the cytoplasm when detecting IgG (Figure S1, CD3).

### Role of the anti-TG2 antibodies in neuronal reactivity of CD patient sera

**Figure 2:**
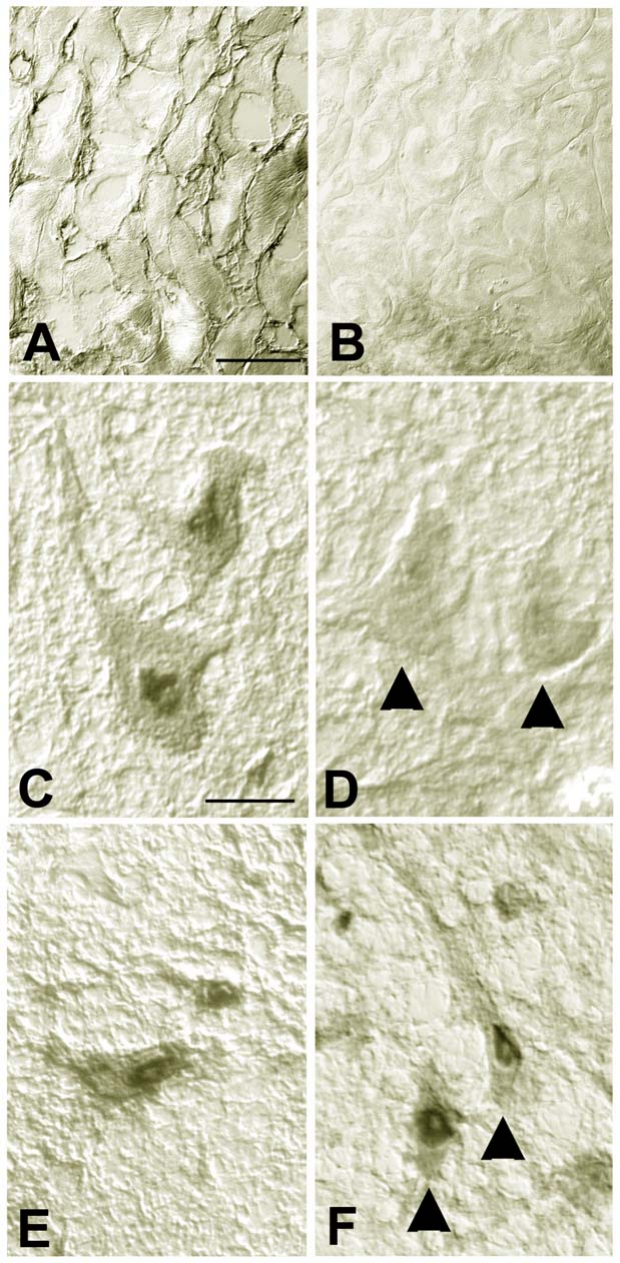
Adsorption of CD serum antibodies on recombinant human TG2 reduces substantially the recognition of neuronal epitopes. Immunohistochemical staining on rat esophagus and brainstem neurons with CD patient serum before (A, C, E) and after depletion of anti-TG2 autoantibodies (B, D, F). Both IgAs (A) and IgGs (not shown) labeled rat esophagus in a pattern reminiscent of that of TG2 distribution in the tissue. The patient's serum also labeled rat neurons in the brainstem (C, IgAs; E, IgGs) with a strong perinuclear staining. After adsorption on recombinant human TG2, no IgA binding to rat esophagus sections could be detected (B). On rat neurons, TG2 adsorption eliminated the IgA reactivity both at the cytoplasmic and nuclear level (D, arrowheads) while the IgG staining was reduced at the cytoplasmic level but remained almost unaltered in the perinuclear area (F, arrowheads). Calibration bars = 20 µm.

Since the development of anti-TG2 autoantibodies is characteristic of CD and neuronal expression of TG2 has been demonstrated [25], we investigated if these antibodies contributed to the anti-neural reactivity detected in CD patient sera. Sera from two randomly selected patients with CD and anti-neural antibodies, were preadsorbed with recombinant h-TG2. Elimination of anti-TG2 antibodies was demonstrated by the absence of staining on rat esophagus sections for both IgA (Figure 2A, B) and IgG (data not shown) with the adsorbed sera. Comparison of the IgA staining on rat brainstem reticular neurons before (Figure 2C), and after adsorption (Figure 2D), showed nearly complete elimination of the reactivity, both at the cytoplasmic and perinuclear level. In contrast, serum preadsorption produced a significant reduction of the cytoplasmic binding of IgG but the perinuclear staining persisted (Figure 2E, F). These data suggest that in patients with CD, anti-TG2 antibodies substantially contribute to the recognition of neuronal epitopes. However, anti-TG2 IgG class antibodies account for only part of the anti-neural IgG-related reactivity.

Since anti-neural IgA reactivity of patients with CD was entirely due to anti-TG2 antibodies, two different types of anti-TG2 scFvs (class1 and 2) were employed that had been affinity selected using recombinant h-TG2 from an IgA phage display library of intestinal lymphocytes of CD patients [8]. These class1 and class2 anti-TG2 scFvs appear to be representative of two distinct patterns of Ig reactivity with TG2 typically seen, i.e. recognize distinct epitopes of TG2 that are commonly targeted in CD [8], [26]. scFvs belonging to class1 and the well characterized anti-TG2 monoclonal antibody CUB7402 recognize human, mouse and guinea pigTG2 whereas class2 scFv reacts only with human-TG2 (Figure S2) [8].

**Figure 3:**
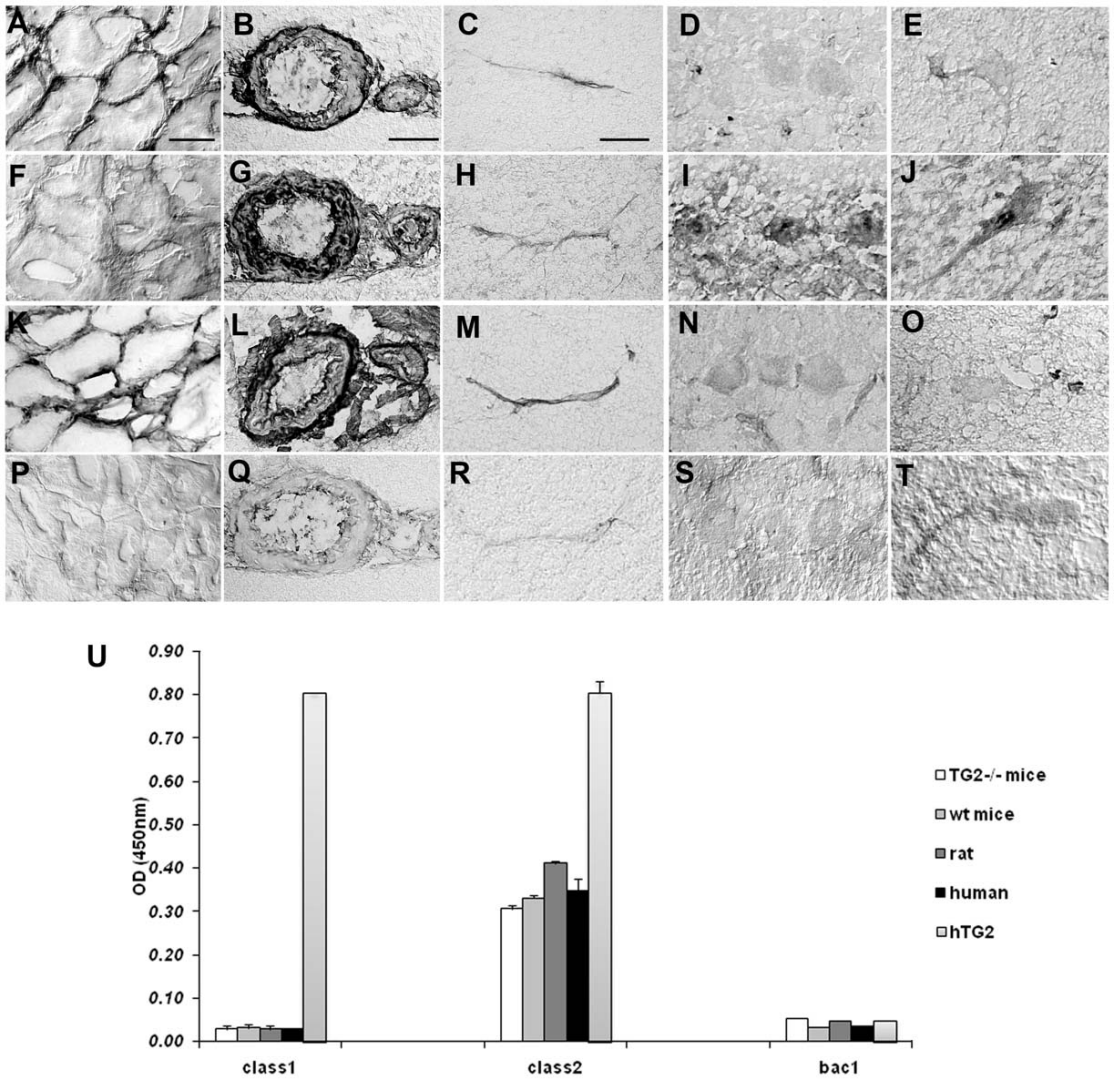
Distinct staining patterns of CD patient derived class1 and class2 IgA scFvs to TG2 on rat esophagus and brain sections. The class1 anti-TG2 scFv strongly labeled rat esophagus (A), with the reticular staining pattern typical for TG2, the tunica adventitia of brain and cerebellum vessels (B) and to a lesser extent brain and cerebellum capillaries (C) but did not recognize brain neurons (D, E). In contrast, the class2 anti-TG2 scFv showed a very faint and diffuse labeling on esophagus sections (F) but strongly labeled the tunica media of brain and cerebellum vessels (G), recognized brain and cerebellar microvessels (H) and also neurons including Purkinje cells (I) and brainstem neurons (J). The monoclonal anti-TG2 antibody CUB7402 reacted on rat tissues with a pattern that is reminiscent of the staining pattern of class1 scFv (K–O). Bac1, a non relevant scFv used as negative control, showed no labeling on rat esophagus (P) brain vessels (Q, R) or neurons (S, T). Calibration bars = 10 µm for C, H, M, R 40 µm for B, G, L, Q and 20 µm for the others. To confirm their specificity, the scFvs were also tested in ELISA on different brain homogenates and on human recombinant TG2. As expected, both class1 and class2 scFvs recognized TG2. However, only the class2 scFv reacted with an epitope in human and rat brain extracts. This epitope was present in brain lysates from wt or TG2^−/−^ mice suggesting that it is not TG2. Bac1 was negative on each substrate used for coating (U).

In immunohistochemistry, class1 anti-TG2 scFv strongly stained rat esophagus, the tunica adventitia of brain and cerebellum blood vessels and the choroid plexus but was negative on neurons (Figure 3A–E). In contrast, class2 showed faint staining on rat esophagus (Figure 3F) but strongly labeled neurons and the tunica media of brain and cerebellum blood vessels with a staining pattern distinct from that of class1 (Figure 3G, I, J). Both anti-TG2 scFvs labeled microvessels in brain (Figure 3C, H) and cerebellum. The antibody CUB7402 gave the same staining pattern as class1 on rat tissues (Figure 3K–O) whereas a control scFv was negative (Figure 3P–T). Identical results were obtained on mouse and monkey brain sections (data not shown) suggesting that the faint staining of class2 scFv on TG2-rich tissues (Figure 3F) is likely to relate to its poor affinity for TG2 of species other than human.

These results were further supported by ELISA experiments, where class2 scFv not only strongly reacted with mouse and rat but also human brain homogenates while the class1 and control scFvs were at background level (Figure 3U). Some reaction of class1 on brain lysates was expected because of its reactivity with vessels in immunohistochemistry (see Figure 3B,C). However, the mild conditions under which the tissue was extracted were likely not able to efficiently solubilise extracellular matrix-bound perivascular TG2.

### Class2 anti-TG2 IgA crossreacts with other transglutaminases

The observed differences in reactivity with tissue structures could relate to selective availability of TG2 epitopes due to alternative splicing [27] or distinct conformations adopted by the protein upon binding of nucleotides or Ca2+ ions, respectively [28] but could also relate to epitopes shared between different types of transglutaminases. Class2 scFv reacted strongly with brain homogenate from TG2−/− mice (Figure 3U) indicating that this antibody can cross-react with an antigen other than TG2. Immunohistochemistry on TG2^−/−^ mouse brain sections confirmed the absence of reactivity with class1 scFv (in blood vessels) while class2 scFv showed a similar anti-neural staining pattern to that seen in wildtype brain (Figure S3).

**Figure 4:**
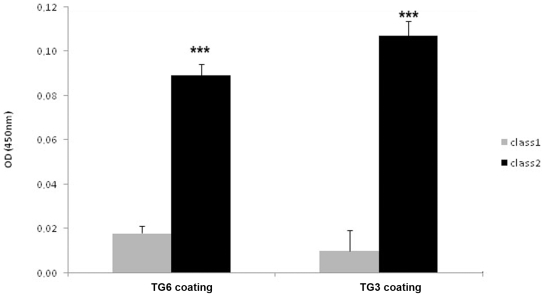
Cross reactivity of class2 but not class1 scFv with different TGases. Anti TG2 scFvs were tested in ELISA on human recombinant TG3 or TG6 as an antigen to determine whether they recognize an epitope that is shared between different TG isozymes. Class2 scFv is reactive with both TG3 and TG6 (P<0,001 *** one way Anova) in contrast to class1 which reacts exclusively with TG2.

As a number of transglutaminases have been detected in brain [24], [27], [29], [30], and all members of the transglutaminase family have a high degree of sequence similarity, we tested whether the scFvs cross reacted with the TG3 and TG6 which are the isoforms most closely related to TG2, both in terms of amino acid sequence and protein activities [31]. Notably TG3 is involved in another gluten sensitive disease (dermatitis herpetiformis) and TG6 is suspected as a possible autoantigen in the neurological manifestations of gluten sensitivity [24]. In ELISA with immobilized recombinant human TG2, TG3 or TG6, class1 scFv reacted only with TG2, while class2 also bound to TG6 and TG3, although to a lesser extent as compared to TG2 (Figure 4). Thus, class1 represents a pure anti-TG2 IgA while class2 scFv turned out to be from an anti-TG2-3-6 IgA.

### Effects of anti-TG2 IgA antibodies on mouse motor coordination

**Figure 5:**
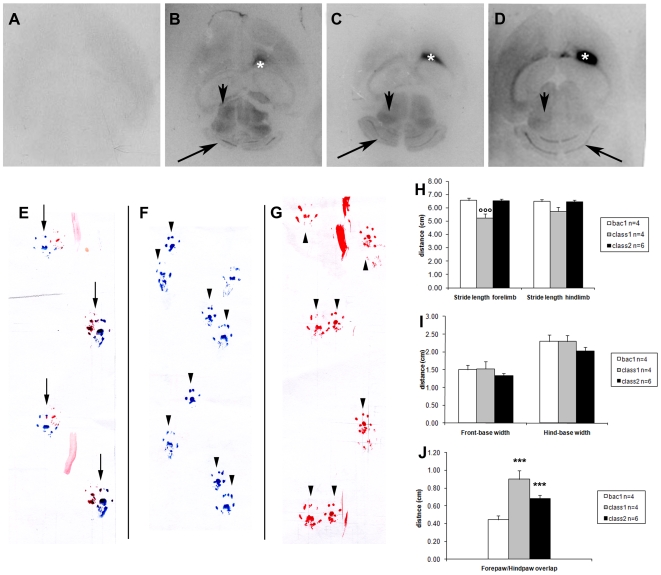
Localization of anti-TG2 scFvs after intraventricular delivery and footprint analysis. The effective delivery of the scFvs after injection, was confirmed on transverse brain sections by detection of the injected biotin conjugated scFvs with streptavidin conjugated horseradish peroxidase. Sections of untreated mice incubated with streptavidin-peroxidase alone showed no discrete labeling (A) whereas sections from mice injected with biotinylated class1 (B), class2 (C) or bac1 (D) scFv clearly demonstrated the presence of these in the lateral ventricles of mice 3 h after injection (asterisks) and in the brain parenchyma even at considerable distance from the ventricles (cerebellum, long arrows; collicoli, short arrows). Mice treated with monoclonal antibodies were tested at 5 h, after antibody injection for gait imbalance with footprint analysis. The R-L overlap clearly demonstrated that the control scFv bac1 had no effect on mice gait (E, arrows, front paws colored with red, back paws colored with blue), on the contrary with both class1 and class2 there was no overlap between left or righ front and back paws (F–G, arrowheads). In particular, four step parameters have been recorded, namely, stride length (H), front and hind-base width (I) and overlap between forepaw and hindpaw placement (J). Mice injected with anti-TG2 monoclonal antibody class1 exhibited a significant shorter forelimb stride length and a less marked hindlimb stride length (H) compared to mice treated with the negative control monoclonal antibody bac1. There was no significant difference between mice injected with class1 or bac1 for the hind and front-base width (I) while mice treated with class1 displayed a greater distance between fore/hindpaw overlap compared to that produced by negative control injected mice (J). Mice injected with monoclonal antibody class2 showed indistinguishable forelimb and hindlimb stride length from those produced by negative control mice (H) a little decrease in the fore and hind-base width (I) while showed a significant different overlap measurement compared to the negative control mice (J). Bars represent the mean value of each measurement considered, n is the number of mice injected with the monoclonal antibodies. Error bars represent SE. **°°°** P = 0,002 one way ANOVA class 1 compared to bac1. *** P<0,001 one way ANOVA class 1 compared to bac1 or class 2 compared to bac1.

We investigated if such anti-TG2 antibodies have a direct causative role in ataxia by injecting the anti-TG2 (class1), the anti-TG2-3-6 (class2) or the control (bac1) scFv into the lateral ventricle of mice and tested their effect on balance using the rotarod test [15]. We demonstrated that mice treated with either class1 or class2 scFv manifested a dramatic motor impairment at 3 h and 6 h after injection, as compared to the preinoculum performance (Figure S4). Symptoms of motor dysfunction disappeared at 24 h post-injection. As expected, the performance of mice treated with the control scFv was not affected (Figure S4). We also tested mice ataxic gait with footprint analysis. R-L overlap demonstrated clear motor impairment in mice treated with anti-TG2 (Figure 5F–G, arrowheads) but not with control scFv (Figure 5E, arrows). In particular mice injected with anti-TG2 class1 exhibited a significant shorter forelimb stride length and a less marked hindlimb stride length (Figure 5H) when compared to mice treated with bac1. No significant difference between mice treated with class1 or bac1 was found for the hind and front-base width (Figure 5I) while mice treated with class1 displayed a greater distance between fore/hindpaw overlap compared to that produced bac1 injected mice (Figure 5J). Mice injected with scFv class2 showed indistinguishable forelimb and hindlimb stride length from those produced by bac1 (Figure 5H), but showed a mild decrease in the fore and hind-base width (Figure 5I) and a significant different overlap measurement compared to bac1-injected mice (Figure 5J).

**Figure 6:**
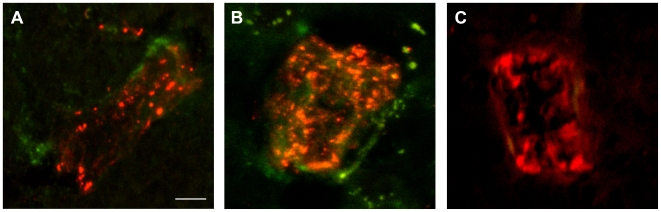
Localization of anti-TG2 scFvs after intraventricular delivery on small brain vessels. Biotinylated scFvs were revealed 3 h after injection by incubating FITC-conjugated streptavidin on brain sections. Brain vessels were stained with anti-von Willebrand Factor (vWF) followed by TRITC conjugated anti-rabbit. Confocal images on sections from mice injected with biotinylated class1 (A) and class2 (B) showed the localization of these antibodies on small brain vessels structures in some cases colocalizing with vWF (class2 B). No presence of bac1 (C) scFv was detected on brain vessels. Calibration bar = 5 µm.

To confirm effective delivery of the antibodies into the lateral ventricles and to investigate their interaction with CNS tissue, biotinylated scFvs were injected, 3 h later brains dissected, and scFvs detected on sections using streptavidin. Horizontal brain sections from untreated mice, incubated with streptavidin-peroxidase alone, showed no labeling above background (Figure 5A). Sections from mice treated with the biotinylated class1, class2 or bac1 scFv clearly demonstrated the presence of the injected scFv within the lateral ventricles (Figure 5B, C, D asterisk), to a lesser extent in the collicoli (Figure 5B, C, D arrowhead), in the cerebellum (Figure 5B, C, D arrow), and also revealed infiltration of the scFv into the brain parenchyma. Since both class1 and class2 scFvs provoked motor impairment in mice and both labeled blood vessels in the brain, we hypothesized that they could bind at the level of the brain vasculature. To determine if the injected scFvs antibodies were able to localize on brain vessels, streptavidin-FITC was used and vessels were stained with anti-vWF revealed with anti-rabbit-TRITC. The vWF is a protein produced by endothelial cells at the inner layer of the blood vessels and is accumulated in storage granules in the cytoplasm of these cells. Confocal images show localization of both class1 and class2 on vessels structures (Figure 6A, B) and confirmed our finding that the two scFv classes have a distinct staining pattern, with class1 binding the more external layers and class2 the more internal layers. No localization of bac1 was detected on vessels (Figure 6C) while it was clearly present in the ventricle (data not shown) as also shown by chemiluminescence (Figure 5D). Haematoxylin and eosin staining of brain sections of each animal demonstrated absence of inflammation (data not shown).

## Discussion

Autoantibodies are a characteristic feature of CD and are thought to contribute to extraintestinal organ involvement within the spectrum of gluten sensitivity. We tested their potential role in the production of neurological deficits and whether a link exists between anti-TG2 antibodies and the neural reactivity of sera.

We found that i) 75% of the CD patient population (without evidence of neurological involvement) has circulating anti-neural IgA and/or IgG antibodies; ii) anti-neural reactivity of serum IgA reflects the presence of anti-TG2 antibodies (belonging to class2), that also cross-react with TG3 and TG6; iii) anti-neural reactivity of serum IgG appears to be determined only in part by the presence of anti-TG2 antibodies; and interestingly, that iv) both TG2-specific (class1) as well as anti-TG2 antibodies crossreacting between TG isozymes (class2) cause ataxia in mice when directly administered to the CNS. This indicates that potentially pathogenic antibodies targeting antigens in the CNS are a common feature among CD patients but that their presence alone is unlikely to be the only event that precipitates into a pathological state. A role for anti-TG antibodies in causing ataxia, however, is strongly indicated by the fact that the control scFv has no effect whereas two different anti-TG2 scFvs produce the same functional deficit in mice. As scFvs do not bear the Fc sequence, these results clearly demonstrate that there is no requirement for complement activation or Fc receptor engagement for induction of motor coordination deficits.

Class1 anti-TG2 recognizes both human and rodent TG2 [8], [26], [32] while the class2 scFv recognizes human but not guinea pig [8], [20], [26] or mouse TG2 [33]. Therefore, it is unlikely that in mice, class2 can exert its effect by reacting with TG2. This is further supported by the fact that class2 scFv reacted with epitopes in the brain from TG2−/− mice and recognized recombinant TG3 and TG6 in ELISA, albeit with lower affinity as compared to human TG2. We therefore hypothesize that cross-reaction with TG3 or TG6 may be responsible for the same pathogenic effect in the animal model.

Although identification of the mechanisms through which anti-TG2 antibodies cause ataxia was beyond the scope of the present study, our findings that both class1 and class2 scFvs labeled cerebrum and cerebellar blood vessels in immunohistochemistry and that they localized on vessels after injection in the lateral ventricle of brain, suggest that these antibodies may cause ataxia through an antibody-antigen interaction at the vascular level. This hypothesis is supported by three recent findings, firstly TG1 [34], TG2 [35], [36] and TG3 [37] are present in large blood vessels and capillaries, secondly, TG1 [34] and TG2 [35] play a role in controlling both blood vessel diameter and permeability, and thirdly, anti-TG2 antibodies from CD patients disturb angiogenesis *in vitro* [38]. Our finding that serum IgA from patients with CD strongly reacted with rat brain blood vessels, is in agreement with a previous study carried out on a smaller group of patients [39]. We have previously shown that such IgA antibodies are deposited in a perivascular distribution in the cerebellum of a patient with gluten ataxia [18]. However, it is not clear at present whether autoantibody accumulation is a consequence of a breached blood–brain barrier or the mediator of such a breach.

The fact that mice show functional deficits rapidly after anti-TG2 scFv administration in the absence of any detectable inflammatory cell infiltration in the CNS suggests an antibody mediated mechanism. Induction of localized vascular permeability is also more consistent with the observed partial reversibility of the functional deficit. However, it remains to be determined whether prolonged antibody exposure can ultimately cause neuronal degeneration.

Anti-TG2 antibodies are found in at least 98% of patients with CD [26], [40] but only a proportion of them develop neurological deficits. Although the reason for this remains unclear, a particularly intriguing result of this study is that the vast majority of CD patients without clinically manifest CNS involvement have circulating antibodies that react with epitopes on neurons. However, while only 20% of CD patients had IgA reactive with neurons, 60% had IgG reactive with neurons. Preadsorption of sera on TG2 revealed a residual IgG reactivity to neurons suggesting that IgG antibodies targeting other neural antigens are present, consistent with previous reports identifying anti-GAD IgGs [41]. Thus, anti-TG antibodies may act in concert with further autoantibodies to cause selective neuronal degeneration.

In conclusion, anti-neural reactivity in CD patients is linked to the presence of class2 anti-TG2-TG3-TG6 antibodies that are able to cause ataxia. Consequently, immunohistochemistry on rat brain sections to identify CD patients with anti-neural reactivity may help identifying a subgroup of patients with a specific risk to develop neurological disorders. However, exposure of the CNS to anti-TG2 that do not cross react with neurons can also cause ataxia. Thus, it remains to be seen whether class2 antibodies are strictly associated with patients with neurological deficits and whether in long-term follow-up the development of such antibodies can be linked to a higher risk of developing neurological complications.

## Supporting Information

Figure S1 This figure represents the most frequent staining pattern of sera from various CD patients characterized by a weak cytoplasmic and a strong perinuclear labeling (CD1 and CD2). Sera from two patients stained only the cytoplasm when detecting IgG (CD3).
(6.16 MB TIF)

Figure S2 Reactivity of monoclonals anti-TG2 on TG2 from various species. Class1 scFv and the commercial CUB7402 recognize human, mouse and guinea pig TG2 whereas class2 scFv reacts only with human-TG2.
(1.86 MB TIF)

Figure S3 Immunohistochemistry on TG2−/− mouse brain sections with patient's serum, class1 or class2 scFv anti-TG2. Immunohistochemistry confirmed the absence of reactivity with class1 scFv (in blood vessels) while class2 scFv showed a similar anti-neural staining pattern to that seen in wildtype brain.
(4.38 MB TIF)

Figure S4 Motor coordination test on the rotarod after intraventricular delivery of anti-TG2 scFvs. Mice treated with monoclonal antibodies were subjected to three rotarod trials (9 rpm) before the intraventricular injection (Pre in.), and were tested at 1 h, 3 h, 6 h, 24 h after antibody injection. The mean latency to fall (maximum trial duration = 120 sec) of the three trials was recorded. Mice treated with anti-TG2 class1 or class2 scFvs exhibited significant impairment of the balance on the rotarod at 3 h and 6 h after injection. Mice treated with the control antibody bac1 showed no decline in performance. (n =  number of animals; error bars represent SEM; ** P<0.01, * P<0.05, Wilcoxon test)
(2.69 MB TIF)

Text S1 (0.03 MB DOC)
