# Supplementary figures and images for: Anti Transglutaminase Antibodies Cause Ataxia in Mice
Source: PLoS One. 2010 Mar 15;5(3):e9698. doi: 10.1371/journal.pone.0009698 (PMC2837746; doi:10.1371/journal.pone.0009698)

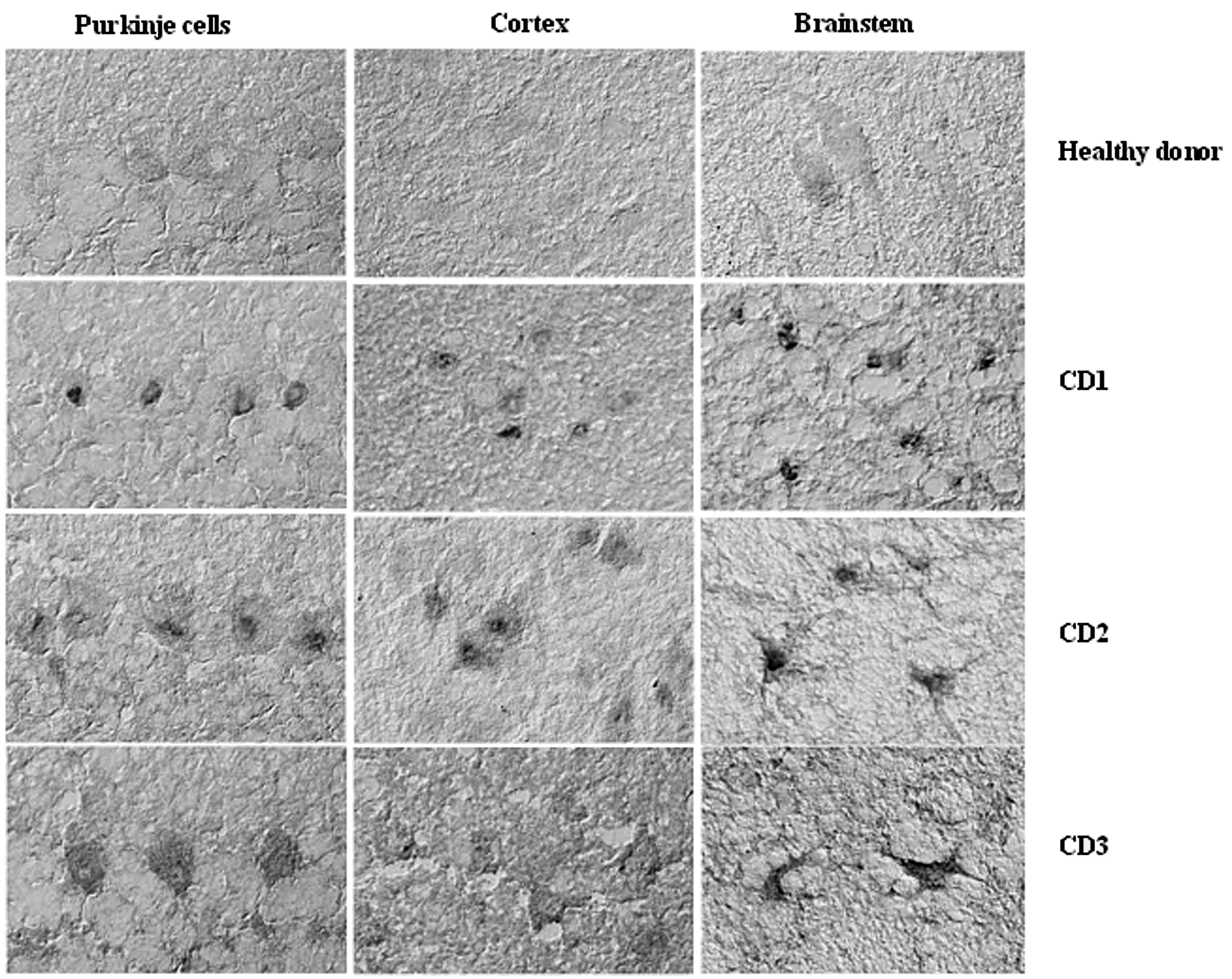

Supplement: Figure S1 — This figure represents the most frequent staining pattern of sera from various CD patients characterized by a weak cytoplasmic and a strong perinuclear labeling (CD1 and CD2). Sera from two patients stained only the cytoplasm when detecting IgG (CD3). (6.16 MB TIF) [file pone.0009698.s001.tif]

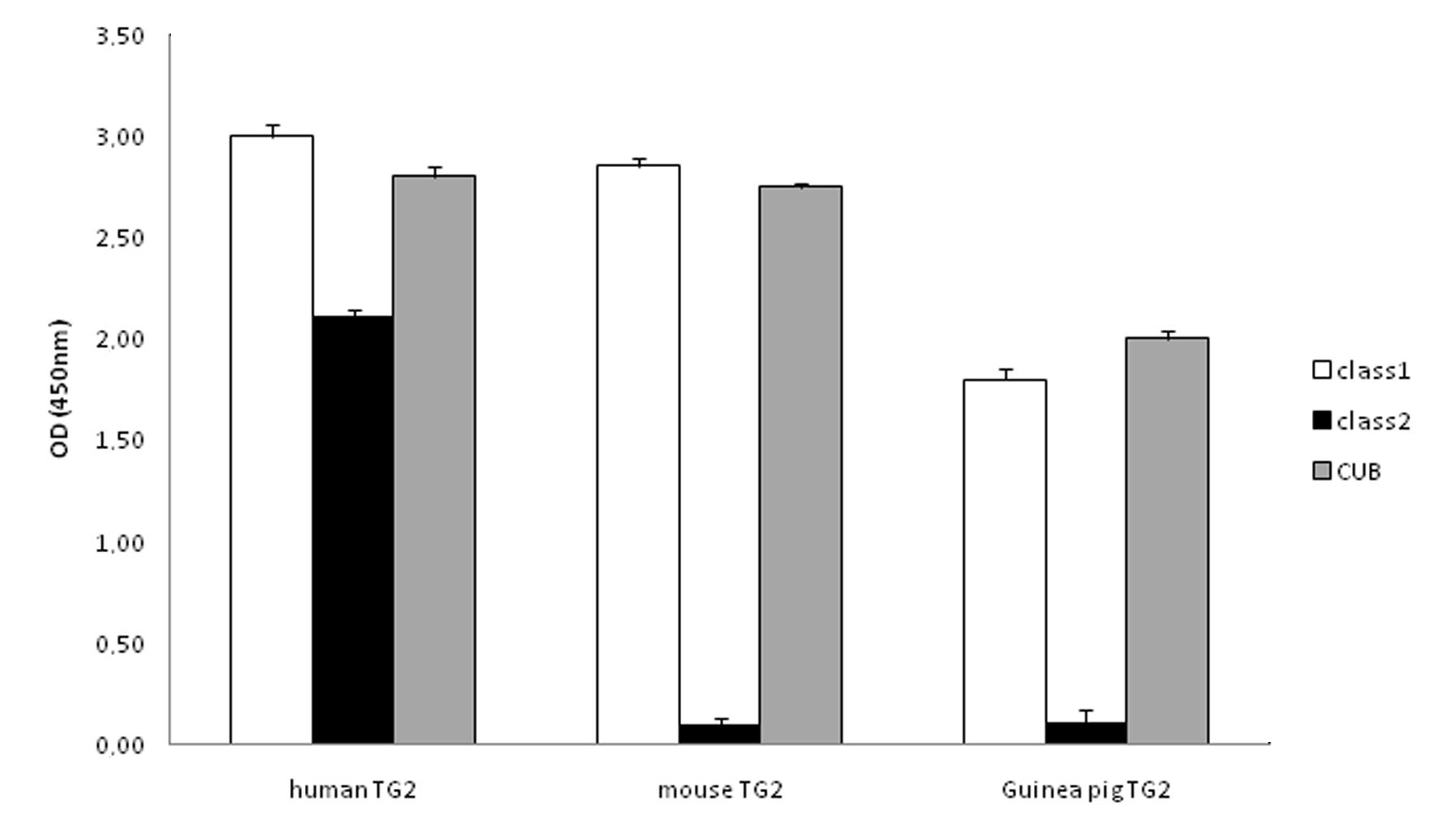

Supplement: Figure S2 — Reactivity of monoclonals anti-TG2 on TG2 from various species. Class1 scFv and the commercial CUB7402 recognize human, mouse and guinea pig TG2 whereas class2 scFv reacts only with human-TG2. (1.86 MB TIF) [file pone.0009698.s002.tif]

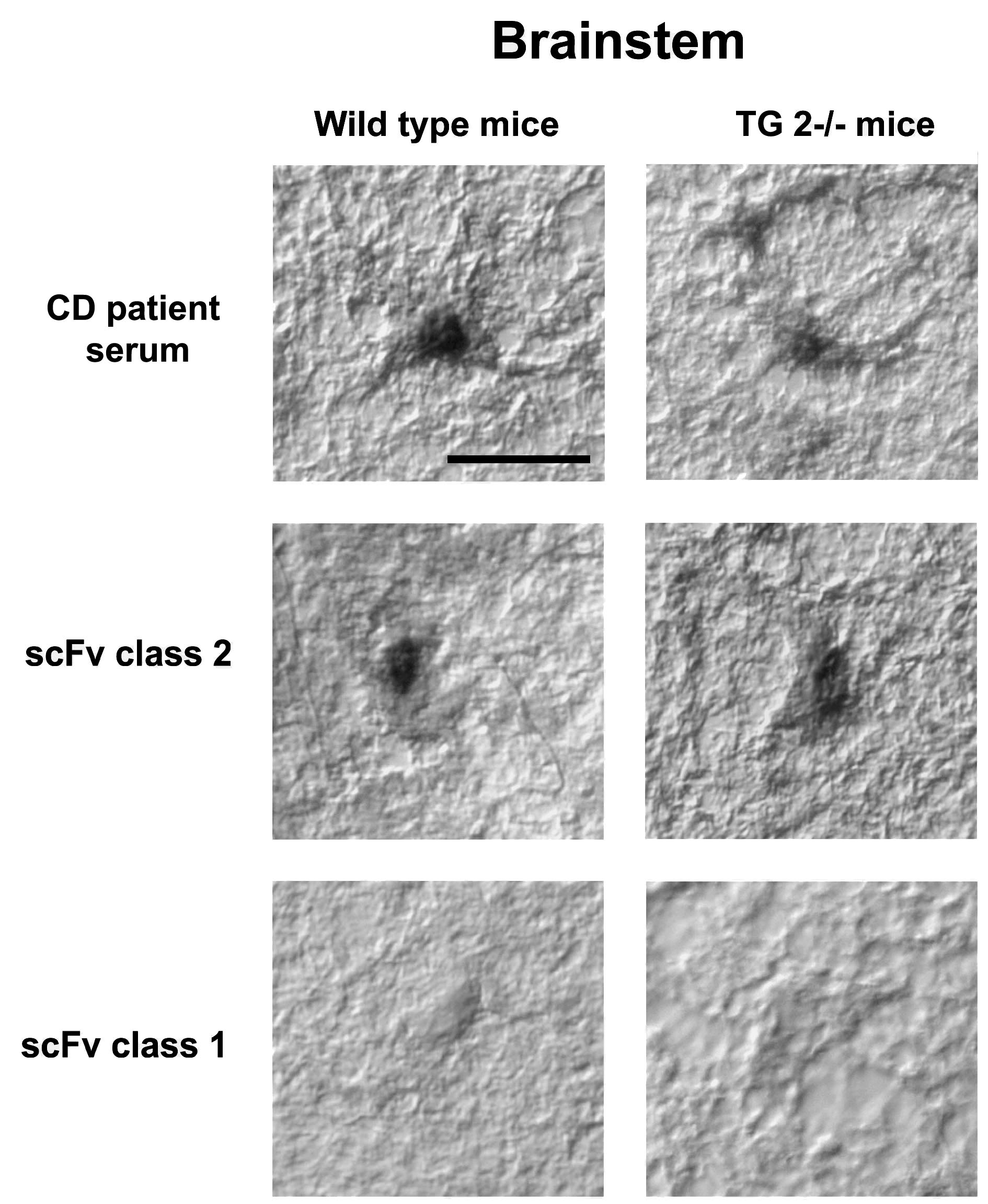

Supplement: Figure S3 — Immunohistochemistry on TG2−/− mouse brain sections with patient's serum, class1 or class2 scFv anti-TG2. Immunohistochemistry confirmed the absence of reactivity with class1 scFv (in blood vessels) while class2 scFv showed a similar anti-neural staining pattern to that seen in wildtype brain. (4.38 MB TIF) [file pone.0009698.s003.tif]

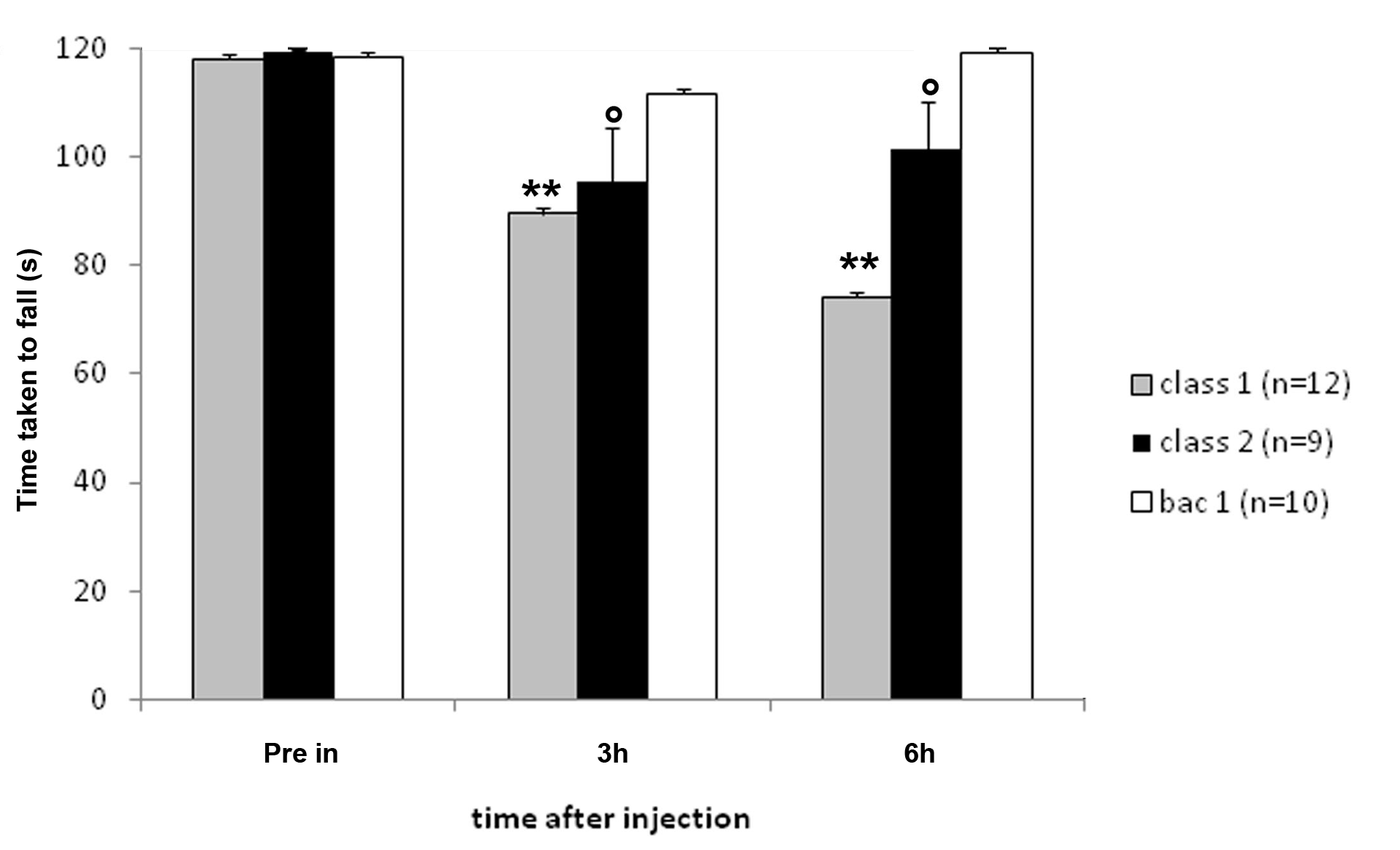

Supplement: Figure S4 — Motor coordination test on the rotarod after intraventricular delivery of anti-TG2 scFvs. Mice treated with monoclonal antibodies were subjected to three rotarod trials (9 rpm) before the intraventricular injection (Pre in.), and were tested at 1 h, 3 h, 6 h, 24 h after antibody injection. The mean latency to fall (maximum trial duration = 120 sec) of the three trials was recorded. Mice treated with anti-TG2 class1 or class2 scFvs exhibited significant impairment of the balance on the rotarod at 3 h and 6 h after injection. Mice treated with the control antibody bac1 showed no decline in performance. (n = number of animals; error bars represent SEM; ** P<0.01, * P<0.05, Wilcoxon test) (2.69 MB TIF) [file pone.0009698.s004.tif]
