# Supplementary material for: Anti Transglutaminase Antibodies Cause Ataxia in Mice
Source: PLoS One. 2010 Mar 15;5(3):e9698. doi: 10.1371/journal.pone.0009698 (PMC2837746; doi:10.1371/journal.pone.0009698)
Supplement: Text S1 — (0.03 MB DOC) [file pone.0009698.s005.doc]

*Motor coordination test*

Adult C57Bl/6 male mice were trained for 3-5 days to maintain balance for 2 min on a rotarod set to rotate at 9 rpm (Ugo Basile, Comerio, Italy). Skill tests were repeated twice a day with three trials per session. After training, mice were anaesthetized with tribromoethanol solution in amyl alcohol and a catheter was implanted in the right lateral ventricle (1 mm right, 0.5 mm anterior to Bregma and 2 mm deep). Mice were re-tested on the rotarod 24h later (preinoculum), then 3µl (100ng/µl in PBS) of scFv were injected intraventricularly over a 3 min time. Mice were tested on the rotarod 1h, 3h, 6h and 24h after the scFv injection and latency to fall was recorded.

To confirm the correct catheter position and that no inflammatory processes had been induced, 25µm horizontal brain sections were collected on gelatine-coated slides and serial sections were stained with hematoxylin and eosin. Data from mice in which the cannula was misplaced were excluded. The final number of animals used for the statistical analysis was 31 (n=10 for bac1; n=12 for class1 and n=9 for class2). The motor coordination tests were performed blind.
